# In Vivo Evaluation of a Pectin-Honey Hydrogel Coating on Polypropylene Mesh in a Rat Model of Acute Hernia

**DOI:** 10.3390/gels7030132

**Published:** 2021-08-31

**Authors:** Cristina Vercelli, Giovanni Re, Selina Iussich, Rosangela Odore, Emanuela Maria Morello, Marco Gandini, Gessica Giusto

**Affiliations:** Department of Veterinary Sciences, University of Turin, Largo Paolo Braccini, N. 2, Grugliasco, 10095 Turin, Italy; giovanni.re@unito.it (G.R.); selina.iussich@unito.it (S.I.); rosangela.odore@unito.it (R.O.); emanuela.morello@unito.it (E.M.M.); marco.gandini@unito.it (M.G.); gessica.giusto@unito.it (G.G.)

**Keywords:** polypropylene, pectin-honey-hydrogel, mesh, adhesion prevention, COX-2, hernia, rats

## Abstract

Investigations about ventral hernia repair are focused on improving the quality, resistance, and biocompatibility of mesh. This study compared plain polypropylene mesh with a pectin-honey hydrogel-coated polypropylene mesh in an acute hernia model in rats. Forty Wistar rats, randomly assigned to two groups, were submitted to laparotomy, and a 1 cm × 2 cm fascial defect was created, centered on the midline. Uncoated (group C) or coated mesh (group T) was inserted in an inlay fashion to repair the defect. After 30 days, the rats were euthanized, and the presence of adhesions to the mesh was macroscopically evaluated. Histology and measurement of COX-2 as tissue inflammation markers were used to assess fascia tissue healing. Grades of adhesion were not different between groups. Histological score and COX-2 expression were not significantly different between groups, except for the higher inflammatory response demonstrated in group T. The pectin-hydrogel coated mesh could not reduce adhesion formation compared to uncoated polypropylene mesh but improved peritoneal regeneration and tissue healing.

## 1. Introduction

Polypropylene (PP) mesh is the most widely used non-degradable synthetic material for abdominal wall defects and hernia repair, considering its tensile strength and flexibility, combined with a low cost [1,2,3,4]. Despite its advantages, this material induces an acute and intense inflammatory reaction that may lead to adhesion formation, fistulation, and chronic abdominal pain [3,5]. Besides being beneficial, they are often associated with complications ranging from seroma to secondary infections. In some cases, complications lead to mesh removal [6,7].

The release of pro-inflammatory cytokines and growth factors is normal during the wound healing process, but an imbalance in early phases can influence collagen deposition by fibroblasts and can compromise the quantity of the fibers, with an inadequate orientation, increasing the risk of hernia [8,9]. Cytokines are synthesized by injured tissue to modulate the inflammatory cascade during the initial response: this concept of general pathology is valid also considering the repair of the abdominal wall [10]. Therefore, tissue engineering modifications of this scaffold may improve its suitability and minimize its side effects. A series of meshes have been developed and tested in several studies. In addition to synthetic meshes, animal-derived materials with appropriate biocompatible features have been investigated, such as bovine pericardium [11], the acellular porcine dermis [12], urinary bladder matrix [13], and acellular analog of humans [14,15]. However, the commercial availability of this type of meshes is limited by the extremely expensive cost of production [13].

Research about new compounds that enhance the performances of meshes is underway. Natural polymers such as gelatin and chitosan have been used as scaffolds for tissue engineering [4,16]. Their high-water content gives these materials the name of hydrogel and permits excellent biocompatibility with several tissues. Furthermore, their elastic properties can minimize inflammatory reactions of the surrounding cells [17,18]. Pectin has been widely used as a scaffold or wound dressing in tissue engineering to improve wound healing and other biomedical purposes due to its biocompatibility, biodegradability, and nontoxicity [16].

The use of honey to enhance wound healing was described by ancient Egyptians and by Hippocrates. At the beginning of the 1990s, it was reported the broad-spectrum antibacterial properties of honey had been attributed to the binding of water and sugars, making them unavailable for microorganisms, the acidity of honey, the production of small amounts of hydrogen peroxide [19]. Honey is composed of several active agents that inhibit the growth of Gram-negative and Gram-positive bacteria [20]. It has anti-inflammatory effects and promotes healing processes [16]. It should be underlined that raw honey is not adequate for medical purposes because it can be contaminated by clostridiospores and/or residues of drugs used in agriculture. For these reasons, deactivation with gamma radiation and sterilization of honey wound dressing is required [19].

In recent years, honey has been used to prevent postoperative peritoneal adhesions [7,11,21,22]. Honey has been used in wound healing since ancient times, and its properties are hygroscopicity (that decreases edema and exerts a barrier effect inhibiting deepening of the wound), lower pH, and hypertonicity (mainly responsible for its antibacterial and antimycotic effect) [22].

Our research group has been focused on research dealing with the wound healing process using pectin-honey hydrogel for several years [7,16,20]. Our previous research demonstrated that pectin-honey hydrogel (PHH) could induce a faster wound healing than liquid Manuka honey or pectin alone if compared to control in rats who underwent a full-thickness excisional wound [16]. Then we successfully investigated the application of PHH to improve healing and to limit adhesion in a rat cecal abrasion model [7]. The PHH meshes used in these studies have been carefully characterized. The biocompatibility was determined thorough complex trials: the hydrogel showed a good water vapor transmission rate and fluid uptake. It was demonstrated to not be cytotoxic in the L929 fibroblast cell line [20]. Moreover, the biocompatibility was confirmed in an in vivo trial in which plasma markers such as interleukin (IL)-1 beta, IL-6, tumor necrosis factor, and prostaglandin E2 were evaluated and did not indicate inflammation or endotoxemia induced by the mesh [20].

Considering our previous experiences and considering that honey has been successfully used to treat infected dehisced hernia wounds [23], we aimed to continue our studies hypothesizing that PHH coating of polypropylene mesh would prevent mesh adhesions and improve fascial healing in a rat in vivo model. Therefore, the objective of the present study was to compare PHH coated and uncoated polypropylene mesh in terms of adhesion formation, tissue healing, and inflammation, using macroscopic, histological, and immunohistochemistry evaluations.

## 2. Results

Among the 40 rats used for the experiment, 3 of the control (C) group and 6 of the treatment (T) group died between postoperative day 1 and day 15. This is considered a normal mortality rate for experimental rats of this age.

**Figure 1:**
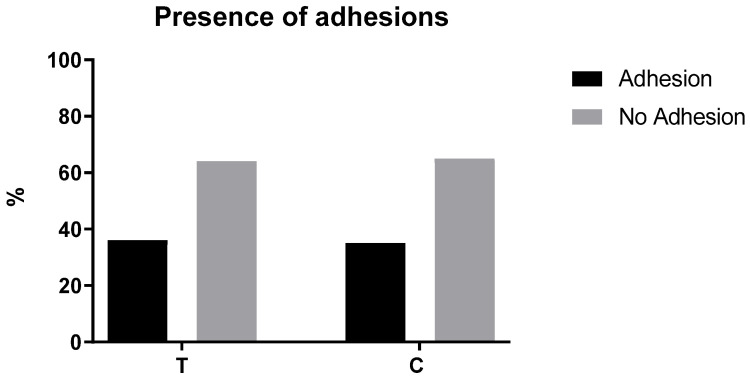
The figure shows the comparison of the percentage of adhesions identified in the control (C) group (*n* = 17) and the treated (T) group (*n* = 14).

In the remaining rats, adhesions between omentum were found in 6 subjects out of 17 (35.3%) of group C and in 5 rats out of 14 in group T (35.7%) (*p* > 0.999, odds ratio = 1.019). Group C rats also showed adhesions between the omentum and the liver, while only 1 rat showed the same lesion in group T. In group C, 8 animals developed adhesion between the distal jejunum and the omentum, while in group T only 1 animal did. In group C, 1 rat developed adhesion between the omentum and the median ligament of bladder. In group T, 3 animals developed adhesions between omentum and linea alba. Results about the distribution of adhesions between groups are represented in Figure 1.

The percentage of coverage (median[range]) of the mesh by the peritoneum was evaluated and showed no differences between groups (100 (30–100)) in group C and 90 (30–100) in group T; *p* = 0.7958).

**Figure 2:**
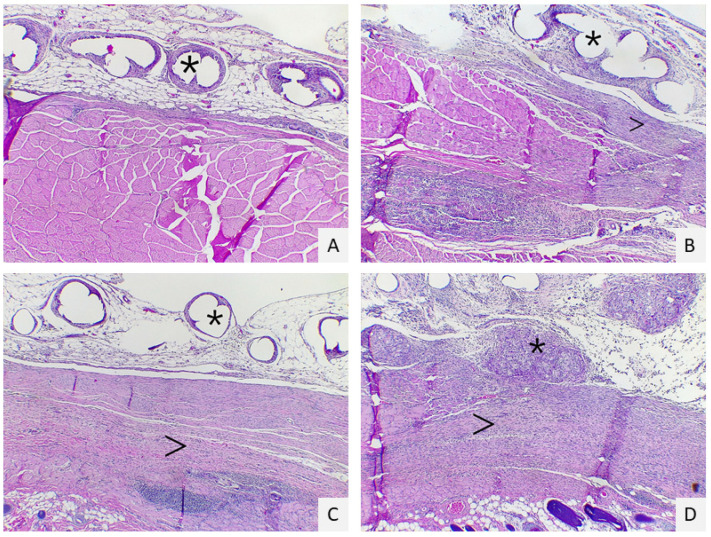
Histological scores of inflammation: score 1 (**A**): multifocal granulomas (asterisk) in adipose tissue with low interstitial fibroplasia (arrow); score 2 (**B**): multifocal granulomas with a moderate amount of fibroplasia score 3 (**C**): multifocal to coalescent granulomas with a moderate amount of fibroplasia; score 4 (**D**): multifocal to coalescent granulomas with a severe amount of interstitial fibroplasia Hematoxylin-eosin staining; magnification: 4×.

Abundant fibroblast proliferation and reactive granulomas induced by the mesh were present in both groups. Still, in group T a significantly higher grade of inflammation at the margin of the granuloma was expressed (*p* = 0.048, *p* = 0.0009, respectively) (Figure 2).

The inflammation in the host tissue and the maturation of the tissue were similar in the two groups, without significant differences (Table 1).

**Figure 3:**
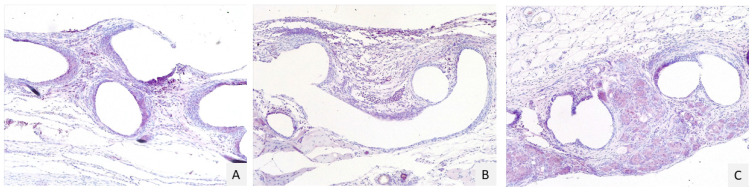
Immunohistochemistry scores to evaluate COX-2 expression. Score 1 (**A**): multifocal and moderate positivity (pink stain) around the granulomas; score 2 (**B**): multifocal and moderate positivity around the granulomas and in interstitial tissue; score 3 (**C**): diffuse and strong positivity around the granuloma and in the interstitial tissue. Magnification 10×.

The median score of immunohistochemistry expression of COX-2 (Figure 3) was 2 in group C and 2 in group T; the median value of the intensity of the COX-2 expression was 1 in the C group and 2 in the group T.

No statistically significant differences were highlighted between the groups (Table 2).

## 3. Discussion

In the present study, the polypropylene mesh coated with PHH did not prevent abdominal adhesion formation but produced a mild improvement in fascial healing, as expressed by a higher number of cell layers and inflammation. However, PHH did not reduce the formation of adhesions involving the mesh. According to the authors’ knowledge, this is the first investigation of host tissue response and adhesion formation comparing PHH coated polypropylene mesh and plain polypropylene mesh in an acute hernia rat model.

Considering the results obtained in the present study of macroscopic evaluation, no dehiscence, purulent material, or abscess were seen. Histological evaluation of the slides stained with H&E confirmed that no infections were present. Moreover, the histological analysis demonstrated an inflammatory process in all samples, but without any significant difference between the two groups 30 days after the surgery. The evaluation of COX-2 in tissue samples collected by all animals demonstrated that no significant differences were observable comparing the two groups.

The inflammatory response induced by meshes seems to be a milestone, and it has to be limited because mesh implant should assist the natural healing process [9]. The authors favorably considered the presence of an inflammatory response since it can be recommendable to induce tissue reaction and to initiate several chemotactic phenomena, principally related to the action of the fibroblast. It has been reported that a strong increase of inflammatory stimuli can induce an excess of fibroblasts proliferation with the inevitable formation of adhesions [24]. In the author’s opinion, in the present study, the formation of adhesion in both groups could be related to some conditions that are hereafter explained. Physical barriers are the most used methods to prevent intra-abdominal adhesions in humans and also in an animal model, but the therapeutic effect of this kind of membrane may be limited to the site of application [25]. We used an inlay approach in which the repair material is posed to the internal aspect of the defect. This method allows greater direct contact of the implant with the abdominal viscera than the onlay approach, which implies that the repair material is attached to the external edges of the abdominal wall defect [26]. The inlay mesh positioning seems to induce a great fixation of the implant with a possible increased risk to induce adhesions [26].

Another hypothesis is that in the present study, adhesions were possibly caused by sutures placed to fix meshes, with or without PHH coating, to the abdominal wall. These sutures were not coated with PHH, and thus, left exposed to the abdominal organ.

The incorporation and the inflammatory reaction in tissue can be predicted according to the pore size, the design of filaments, and their spatial distribution. It has been described that polypropylene meshes with large pore size and low weight can reduce fibrosis formation and persistency [27]. In the present study, we used macroporous polypropylene (Bard^®^ Mesh-BD, Franklin Lakes, NJ, USA) that has been described as a resistant material, able to avoid dehiscence in the period after the surgery (30 and 60 days) if compared to other meshes with different weights and pore size [27]. Moreover, the large pore size permits the penetration of fibroblast that can assure proper incorporation of the mesh [27]. Therefore, the authors cannot exclude that the presence of adhesions can be due to the penetration of fibroblast. It will be necessary to evaluate the coating of other meshes, such as Ultrapro^®^ (Ethicon, Raritan, NJ, USA) that has been defined as the best material in the healing process of the abdominal wall [27].

Polypropylene mesh triggers an elevated short-term reaction with discomfort for patients. It can result in a higher collagen density in the host scar tissue that can remain in a long postoperative period [24]. To carefully evaluate the mesh implants over time, several biopsies of the abdominal wall would be necessary, leading to unacceptable ethical issues. In humans, the evolution could be monitored with computer tomography or magnetic resonance [28]. Pascual et al. [29] observed that an intense inflammatory response is related to the presence of absorbable material and a low expression of growth factors, worse collagen deposition, and worse mesh integration [29]. Moreover, hydrogel-coated meshes seem to induce a small tissue reaction compared to normal polypropylene meshes [30,31].

Honey stimulates cytokines production and promote lymphocytes (B and T) migration. These two factors enhance wound-edema reduction, increase tissue perfusion, oxygenation, fibroblast, and angiogenesis proliferation [23,32,33]. The stimulation and promotion of the wound healing process induced by honey have been confirmed by comparing its effects with those produced by silver dressing [34].

In the present study, the well-known property of PP meshes was added to those of pectin and honey, forming an innovative hydrogel. This material is easy to handle and has low-cost production and intrinsic antibacterial activity [16,20].

These experiments commonly involve rats because the rat abdomen has a similar anatomic structure compared to humans. Both have four abdominal wall muscles, the same muscle fibers orientation, and a similar length and strength of muscle excursion [34]. For all these reasons, the present in vivo model was chosen. The observational period of 30 days after surgery was chosen because it corresponds to approximately 3 years in humans, representing a sufficient time for evaluation of scar remodeling [35]. Rats represent an easy to handle, reproducible and inexpensive model compared to other animal models (such as sheep, pigs, dogs) that can investigate ventral abdominal hernia repair. Moreover, the smaller overall body surface area permits the evaluation of materials that are experimentally in nature and could not be abundant [36]. However, the translation to humans might be limited because rats are genetically standardized, quadruped, and lack the typical co-morbidities that can affect humans (i.e., diabetes, connective tissue defect, and obesity) that can easily lead to abdominal wall defect. Moreover, it is difficult to compare the results obtained by the different investigations because no consensus exists about mesh classification and nomenclature [9,24] and about the surgical procedure in animal models [37].

Our method could be reliable to demonstrate that PHHs honey-coated is not useful to avoid adherence formation in this site. Further studies are needed to verify if the same coating on other types of meshes could reach this important goal.

## 4. Conclusions

The present study aimed to compare the effects of PP meshes, coated and uncoated with pectin-honey- hydrogel in an in vivo acute hernia rat model. The aim was to perform several evaluations using a complex approach consisting of a macroscopic inspection, histology, and immunohistochemistry. According to the authors’ knowledge, this is the first study reporting this kind of evaluation. The assays demonstrated that the PP mesh coated with PHH permitted excellent results in peritoneal regeneration while being poor in adhesion prevention. The new biomaterial can induce a rapid regeneration of tissue within the mesh, confirming the beneficial effect of honey in the wound healing process. However, further investigations have been hypothesized to evaluate if other meshes can be coated with PHH to prevent or avoid adhesion formation.

## 5. Materials and Methods

Forty Wistar male rats weighing 225–250 g (purchased at Envigo, Milan, Italy) were used to produce an acute hernia model and randomly assigned to two groups, according to the mesh used to repair the hernia defect.

### 5.1. Preparation of Pectin-Honey Hydrogels (PHHs) and Mesh Coating

A modified version of the preparation method described by Walker [38] was used, according to Giusto et al. [16]. Briefly, the PHHs were prepared from a starting solution (1:1 *v*/*v*) of liquid honey and sterile deionized water. Powder pectin (Sigma-Aldrich, Milan, Italy)) was then added gradually (0.5:1 *w*/*v*) and continuously stirred to homogenize the mixture. The resulting foam was spread onto 10 cm × 10 cm polypropylene meshes (Bard^®^ Mesh-BD, Franklin Lakes, NJ, USA) with a standard thickness of 2 mm and hot-air-dried at 40 ± 0.5 °C for 6 h. The coated mesh was cut into 2 cm × 3 cm pieces and further conditioned in an air drier at 25 ± 1 °C for 5 days. The PHH-coated meshes were then collected and vacuum packed in polyethylene bags before being sterilized by gamma-irradiation at 25 kg grays (kGs) (Sterigenics International LLTC, Bologna, Italy).

### 5.2. Surgical Procedures

All procedures were approved by the Bioethical Committee of the University of Turin and by the Italian Ministry of Health (protocol number 262/2015-PR on 20 April 2015).

Each rat was housed in a single cage for 7 days before surgery. The room temperature was set at 23 °C, and the cages were cleaned daily. The rats were fed with a commercial diet and water was provided *ad libitum*.

Anesthesia was induced by intramuscular administration of 5 mg/kg of xylazine (Rompum, Elanco Italia S.p.A, Firenze, Italy) and 50 mg/kg of tiletamine and zolazepam (Zoletil, Virbac S.r.l., Milan, Italy). The abdomen was shaved, and the skin was aseptically prepared.

A 4 cm midline incision was made in the skin and subcutaneous tissue. A full-thickness 1 cm × 2 cm abdominal defect was created. The animals were randomly assigned to the experimental groups of 20 rats, each using a free online calculator (www.random.org, accessed on 23 October 2019).

In group C, plain 2 cm × 3 cm pieces of polypropylene mesh (PP) (Bard^®^ Mesh, Franklin Lakes, NJ, USA) as inserted in an inlay fashion to repair the defect. In comparison, a 2 cm × 3 cm pieces of PHH-coated polypropylene mesh were applied in group T using the same technique.

PP mesh and PP mesh coated with PHH were fixed on the edges of the defect using interrupted suture with 4-0 USP polypropylene (Covidien, Segrate, Milan, Italy). The skin was closed with 3-0 USP nylon (Covidien, Segrate, Milan, Italy). The procedure lasted one hour, rats were placed on a warm plate to avoid hypothermia, and 5 mL isotonic sodium chloride solution (Sodio Cloruro 0.9%, Galenica Senese, Monteroni d’Arbia, Siena, Italy) was administered subcutaneously.

### 5.3. Macroscopically Examination

Thirty days post-surgery, animals were anesthetized using 5 mg/kg of xylazine (Rompum, Elanco Italia S.p.A, Firenze, Italy) and 50 mg/kg of tiletamine and zolazepam (Zoletil, Virbac S.r.l., Milan, Italy), and then euthanized by atlas-occipital dislocation. The abdominal cavity was inspected through a U-shaped incision in the anterior part of the abdominal wall. Any macroscopic adhesion in the abdominal cavity, between the mesh and organs or among organs, was identified and recorded.

### 5.4. Histopathological Evaluation

The abdominal walls were collected for histopathological examination and fixed in a 10% formaldehyde buffered solution. Samples were routinely processed by dehydration and paraffin embedding, and 3 µm cross-sections were cut. The samples were examined under a light microscope after hematoxylin-eosin staining and evaluated blindly by an expert pathologist to measure the coverage of the mesh by the peritoneum, tissue adhesions, inflammation response in the host tissue, and tissue maturation. All materials were purchased by Sigma-Aldrich (Milan, Italy). Results were expressed as scores, as previously recorded by Pereira-Lucena and colleagues [9] and summarized in Table 3.

### 5.5. Immunohistochemical Analysis

The immunohistochemistry was performed on 4 µm sections of formalin-fixed, paraffin-embedded tissues. Endogenous peroxidase activity was blocked with 3% hydrogen peroxide in methanol for 30 min at room temperature. Sections were exposed to high-temperature antigen unmasking by incubation at 98 °C with citric acid buffer, pH 6.0. Tissue sections were incubated for 1 h at room temperature with cyclooxygenase 2 antibody, diluted 1:100 (COX-2—rats polyclonal antibody, ab15191, Abcam, Cambridge, UK), and revealed with the Vector VIP Substratekit for peroxidase (Vector Laboratories, Burlingame, CA, USA). To objectively evaluate the tissue reaction and expression of COX-2, the scoring scales presented in Table 4 were applied [9].

### 5.6. Statistical Analysis

Data analysis was performed using Prism 9.0 software (Graph Pad, San Diego, CA, USA). The mesh coverage by the peritoneum was expressed as median and upper and lower confidence interval and compared with the Mann–Whitney test. The presence of adhesions in the two groups was compared using a contingency table applying Fisher’s exact test. Data derived from the margin of granuloma inflammation induced in the host tissue by the mesh, tissue maturation, and evaluation of COX-2 expression were analyzed using the Shapiro–Wilk normality test and Mann-Whitney test. A significant value was stated at *p* < 0.05. Data are expressed as a median and upper and lower confidence interval.

## Figures and Tables

**Table 1:** Comparison of histological scores (Mann-Whitney test).

	C (*n* = 17)	T (*n* = 14)	*p* Value (<0.05)
Cells layers at margins of the granulomas	2 (1–4) 1.55–2.45	3 (1–4) 2.135–3.00	0.0488
Inflammatory reaction in the host tissue	3 (1–4) 1.98–3.19	2.5 (1–4) 1.96–3.04	0.7869
Inflammatory response on the mesh surface	2 (2–4) 2.04–2.66	4 (2–4) 2.94–3.92	0.0009
Tissue maturation	2(1–3) 1.34–2.19	2.5 (1–4) 1.60–2.82	0.2538

**Table 2:** Expression of COX-2 and intensity of the positivity.

	C (*n* = 17)	T (*n* = 14)	*p* Value (<0.05)
% cells	2 (1–3) IQR: 1.45–2.2	2 (1–3) 1.9–2.64	0.076
Intensity	1 (1–2) 1.1–1.84	2 (1–3) 1.45–2.12	0.1153

**Table 3:** Numeric scales to evaluate tissue inflammatory response in groups (Pereira-Lucena et al., 2014) [9].

Score	Cells Layers at the Margins of the Granulomas	Inflammatory Reaction in the Host Tissue	Inflammatory Response on the Mesh Surface	Tissue Maturation
1	1–4 layers	Non-dense, mature fibrous tissue	Fibroblast without macrophages or foreign body cells	Dense, mature interstitial tissue, similar to a normal connective or adipose tissue
2	5–9 layers	Immature fibrous tissue with fibroblasts and little collagen	Isolated foci or macrophages or foreign body cells	Interstitial tissue with blood vessels, fibroblasts, and a few macrophages
3	10–30 layers	Dense granular tissue with fibroblasts and many inflammatory cells	One layer of macrophages foreign body cells	Interstitial tissue with giant inflammatory cells but with permeating connective tissue
4	>30 layers	Mass of inflammatory cells, with disorganized connective tissue	Multiple layers of macrophages and foreign body cells	Mass of inflammatory cells without permeating connective tissue

**Table 4:** Numeric scales to value COX-2 immunohistochemistry expression in groups (Pereira-Lucena et al., 2014) [9].

Score	% Cells	Intensity
1	0–25	Weak
2	26–50	Moderate
3	51–100	Strong

## Data Availability

The data presented in this study are contained within the article.
